# Methodology for optimizing quadrat size in sparse vegetation surveys: A desert case study from the Tarim Basin

**DOI:** 10.1371/journal.pone.0235469

**Published:** 2020-08-26

**Authors:** Li Hao, Shi Qingdong, Bilal Imin, Nijat Kasim

**Affiliations:** 1 College of Resources and Environment Science, Xinjiang University, Urumqi, China; 2 Institute of Arid Ecology and Environment, Xinjiang University, Urumqi, China; 3 Key Laboratory of Oasis Ecology, Xinjiang University, Urumqi, China; 4 College of Biology and Geography Sciences, Yili Normal University, Yining, China; Areospace Information Research Institute Chinese Academy of Sciences, CHINA

## Abstract

Random sampling is an important approach to field vegetation surveys. However, sampling surveys in desert areas are difficult because determining an appropriate quadrat size that represent the sparse and unevenly distributed vegetation is challenging. In this study, we present a methodology for quadrat size optimization based on low-altitude high-precision unmanned aerial vehicle (UAV) images. Using the Daliyaboyi Oasis as our study area, we simulated random sampling and analyzed the frequency distribution and variation in the fractional vegetation cover (FVC) index of the samples. Our results show that quadrats of 50 m × 50 m size are the most representative for sampling surveys in this location. The method exploits UAV technology to rapidly acquire vegetation information and overcomes the shortcomings of traditional methods that rely on labor-intensive fieldwork to collect species-area relationship (SAR) data. Our method presents two major advantages: (1) speed and efficiency stemming from the application of UAV, which also effectively overcomes the difficulties posed in vegetation surveys by the challenging desert climate and terrain; (2) the large sample size enabled by the use of a sampling simulation. Our methodology is thus highly suitable for selecting the optimal quadrat size and making accurate estimates, and can improve the efficiency and accuracy of field vegetation sampling surveys.

## 1. Introduction

Random sampling survey is one of the most important methods for conducting ecological research; it is widely used in studies of plant species diversity, species association analysis, vegetation spatial pattern research, biomass estimation, and other areas [1–3]. In field vegetation surveys, especially in large-scale comprehensive surveys, the use of a single quadrat size is both convenient and effective; this approach is thus widely used in field vegetation surveys [4, 5].

The concept of spatial scale is among the most fundamental concepts in ecological research, making quadrat size a vital component of field survey design [6–8]. No model can adequately explain the details of an ecosystem if it ignores spatial scale; such an oversight can introduce wrong characteristics into the system description [9, 10]. Sampling surveys can obtain detailed quantitative descriptions of vegetation characteristics, but an unsuitable sampling design can significantly limit the validity and usefulness of field survey data [11]. Generally, high-frequency and low-variance sampling approaches are considered the most representative in statistics [12]. Quadrat size affects the frequency distribution and the variance of samples, and thus has an impact on whether the sample is considered representative [13]. Different quadrat sizes result in different sample characteristics and different population estimates [14].

As a result of spatial heterogeneity, any adjustment of the quadrat size in a given study design strongly modifies the variance of the samples. Some studies have shown a nonlinear relationship between quadrat area and sample variance. Research on pasture crops shows that increasing quadrat area can effectively suppress the variance of samples, reducing it by 68% or more [12]. Another study also showed that the use of large quadrats can compensate for the spatial heterogeneity of a small area [10]. Although enlarging quadrat size can reduce the variance of the samples, a larger quadrat area may not be a better sampling design [15]. It is important to determine the optimal quadrat size based on the features of the study area and the limitations imposed by climate, terrain, and available labor [16]. Although this is an important issue, research on the optimal quadrat size is scarce.

Currently, most methods use the frequency of species as the key indicator for determining the appropriate quadrat size, but this method is not without its limitations, particularly in desert areas. The species-area relationship (SAR) is one of the general methods; when the SAR is used, the species-area curve is drawn on the basis of field survey data, and the inflection point of the curve is taken as the quadrat size. It is generally believed that a quadrat should cover 63%–86% of the species in the survey area; for areas with fewer species, some researchers consider 20% to be a reasonable lower limit [17]. However, the application of the SAR method in desert areas has a few limitations: (1) additional fieldwork is required to collect data for drawing the SAR curve, which is problematic due to the difficulty of conducting fieldwork in such challenging locations; (2) as a rule, desert vegetation is distributed in patches and has high spatial heterogeneity [5], which increases the variation in the samples and results in reduced robustness of the sampling surveys [8, 18]; (3) the occurrence of extrema may present a problem. Overall, deserts have fewer plant species than other habitats and their clustering can skew the calculation and result in a quadrat size that is too large or too small.

Furthermore, *Populus euphratica* is an important dominant desert species, widely distributed in Xinjiang, north-western China; it plays an indispensable role in protecting oasis ecosystems [19]. Several studies on *Populus euphratica* populations have been carried out on the basis of field-measured data, but the basic and important issue of choosing the optimal quadrat size for field surveys has remained unaddressed. Frequently, the choice of quadrat size relies on the subjective experiences of the researchers, and the question of whether it is appropriate is rarely asked. This can result in the negative effects of an inappropriate quadrat size on sampling results being ignored.

Fractional vegetation cover (FVC) is a comprehensive parameter used to characterize the extent of land surface vegetation cover [20–22]. FVC is especially useful in desert areas, where it is often used as an effective indicator of desertification due to its ability to reflect spatial vegetation patterns. Thus, from an ecological perspective, the adoption of the FVC index is useful in desert areas for determining the optimal quadrat size for vegetation surveys.

In summary, the sampling design of vegetation surveys in sparse desert areas faces difficulties in determining the appropriate quadrat size. Our study aimed to solve this problem by proposing a new methodology for quadrat size optimization in desert areas. Our methodology consists of three steps: (1) simulating random sampling via the Monte Carlo method using low-altitude unmanned aerial vehicle (UAV) images to establish sample sets; (2) calculating the FVC of the sample sets; and (3) analyzing the frequency distribution and the variation of FVC to determine the optimal quadrat size.

## 2. Materials and methods

### 2.1 Study area

We selected the Daliyaboyi Oasis as our study area, located in Xinjiang Province in the northwest of China (38°16′–38°37′N, 81°05′–81°46′E), as it is representative of a desert ecosystem [23, 24]. After flowing more than 200 km through the Taklimakan Desert, the Keriya River, originating in the south of the Kunlun Mountains, forms the natural Daliyaboyi Oasis. The core area of the oasis is 324 km^2^ [25], the annual average precipitation is less than 10 mm [26], the weather is dominated by dust storms and floating dust, the vegetation composition is dominated by *Populus euphratica* and *Tamarix chinensis* [23]. Due to the lack of industrial and agricultural activities, the oasis is very rarely disturbed by human activities [27]. The study area is our field observation and research station for desert vegetation, and no permission is required to enter the field site to perform field vegetation surveys.

### 2.2 Collection and preprocessing of UAV images

Data were collected in July 2018, using the DJI Phantom 4 UAV. The heading and lateral overlap ratio was set to 80%, the flight height was approximately 60 m, and the imaging focal length was 4 mm. The Pix4D software was used for correction and stitching processing. After stitching the images, the spatial resolution was 0.026 m.

### 2.3 Simulation of random sampling

**Fig 1:**
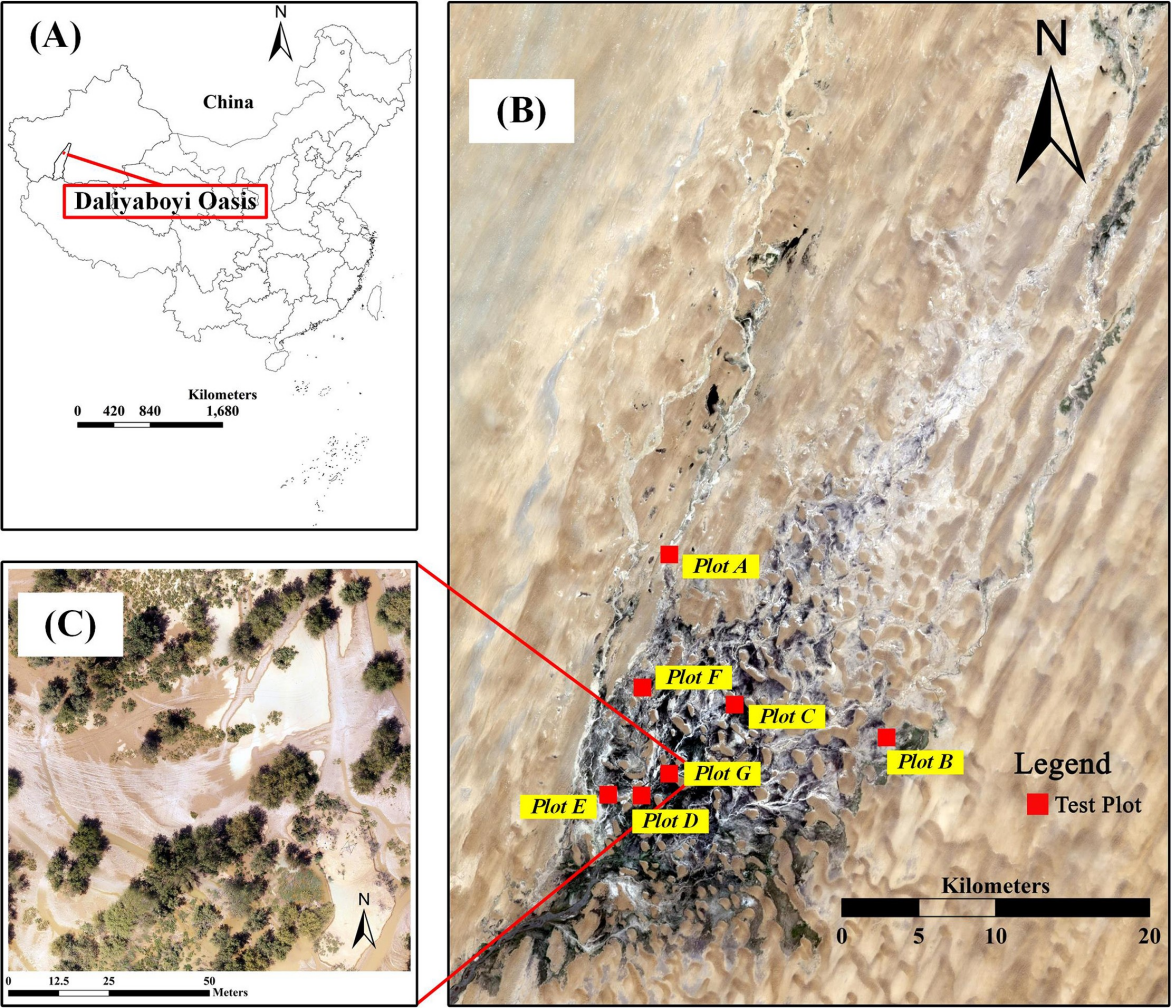
The study site. (A) The study site location: Daliyaboyi Oasis, Yutian county, Xinjiang Province, China. (B) Test plot locations. The background image is a Landsat OLI scene acquired on July 23, 2018 and cropped to our study area, presented with a band combination of 4/3/2. (C) Unmanned Aerial Vehicle (UAV) image of plot G. The image was acquired using a DJI Phantom 4 UAV on July 19, 2018 and preprocessed with Pix4D.

**Fig 2:**
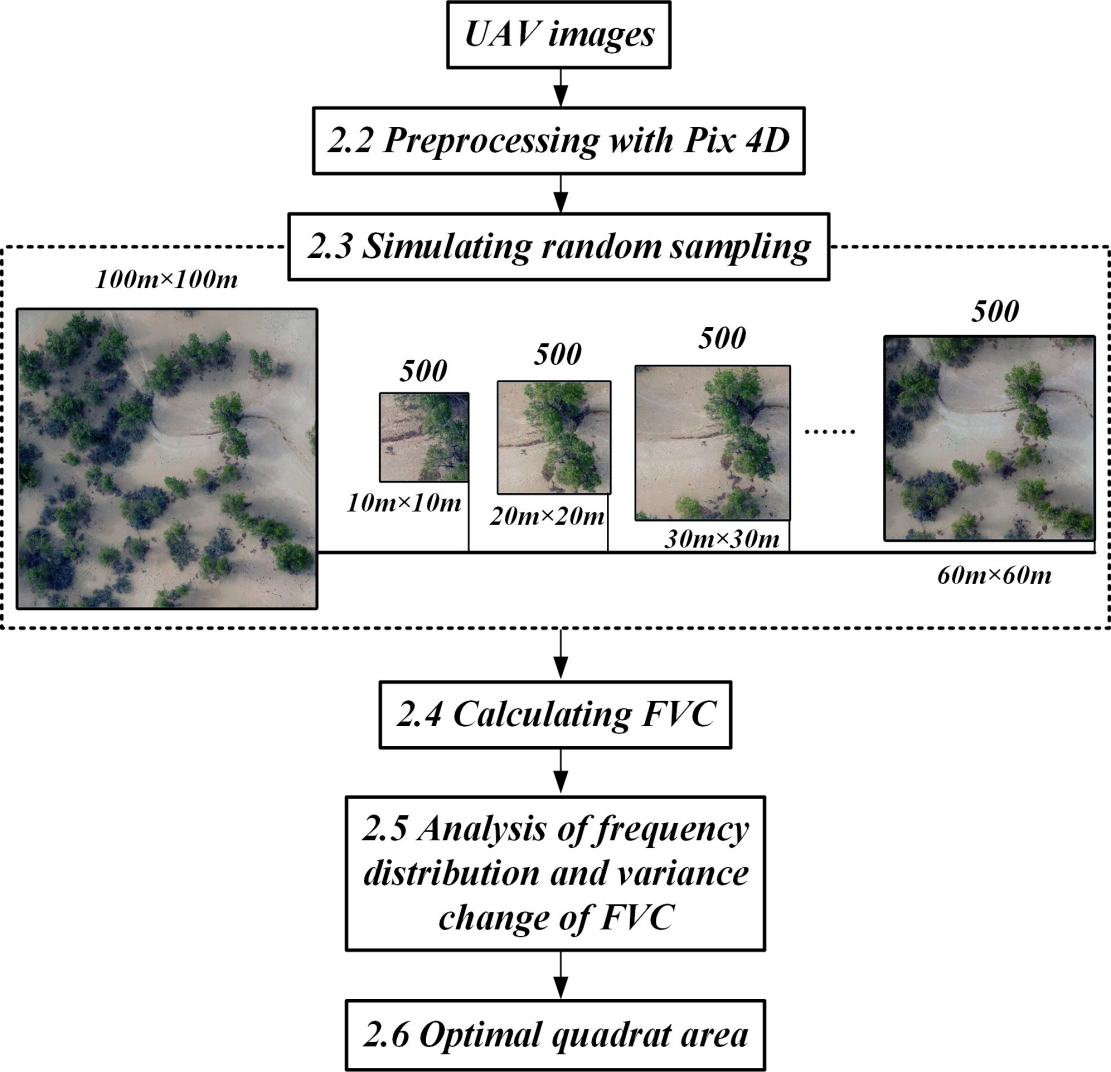
Flowchart of the study methodology. A software named *Extract* is used to repeatedly (500 times) randomly crop a fixed-size (quadrat size) subplot from the test image to simulate random sampling.

Generally, the quadrat size used in field vegetation surveys is less than 100 m × 100 m. Therefore, we selected seven typical 100 m × 100 m plots for the random sampling simulation (Fig 1). The simulation of random sampling was performed by cropping a number of fixed-size pictures from the UAV images of the test plots. We used the cropping tool *Extract* with the cropping method set to the random mode; this causes the software to apply the Monte Carlo method to generate random numbers that determine the cropping positions. In this procedure, the process of random cropping can be regarded as a random sampling process, and the size of the cropped pictures can be regarded as the quadrat size, while the number of pictures obtained by cropping is the sample size (Fig 2).

We had seven UAV images (100 m × 100 m) that corresponded to seven different test plots (Fig 1). For each test plot, six sample sets were created with the following quadrat sizes: 10 m × 10 m, 20 m × 20 m, 30 m × 30 m, 40 m × 40 m, 50 m × 50 m, and 60 m × 60 m; the sample size of every set was 500.

### 2.4 Calculation of the sample FVC

Thereafter, we calculated the FVC under different quadrat sizes and counted the distribution frequency. We used the vegetation index threshold method with IDL software version 8.5 to calculate the FVC of the samples in batches.

There are various methods for estimating the FVC from low-altitude high-precision UAV RGB images; we chose the vegetation index threshold method due to its clear meaning and simple steps. Low-altitude UAV images have a high spatial resolution that can reach sub-centimeter levels. There are very few mixed pixels in the images, so they can be regarded as pure pixels; this means that a single pixel only belongs to one ground object category—either vegetation or non-vegetation [28]. Therefore, the FVC can be quickly estimated using the threshold method to distinguish vegetation pixels and non-vegetation pixels after calculating the vegetation index with UAV images. In addition, the vegetation index threshold method is simple and effective as it is highly automated. The IDL software can be used to calculate the FVC in batches, which meets the study demands when large quantities are required.

#### 2.4.1 Threshold segmentation method

The threshold segmentation method known as the Otsu method (named after the Japanese researcher Nobuyuki Otsu) is widely used in image processing for cluster-based image thresholding, which converts a gray level image to a binary image via the maximization of inter-class variation [29]. It is considered an effective and automatic image segmentation algorithm [30].

Assuming that the grayscale image contained a number of gray levels (denoted by L), a threshold (denoted by *t*) separated the pixels into either foreground or background categories. The foreground pixels accounted for a certain percentage (w_0_) of the image, and the average gray level for a foreground pixel was denoted by u_0_; the background pixels accounted for w_1_ percent, and the average gray level for a background pixel was u_1_. The overall average gray level for the image was denoted by u, and we used *g* to represent the inter-class variance.

The relationships between these values were as follows:

$$u=w_{0}\times u_{0}+w_{1}\times u_{1}$$ (1)

The inter-class variance was calculated as:

$$g=w_{0}{\left(u_{0-}u\right)}^{2}+w_{1}{\left(u_{1-}u\right)}^{2}$$ (2)

From Eqs (1) and (2) we obtained:

$$g=w_{0}w_{1}{\left(u_{0-}u_{1}\right)}^{2}$$ (3)

Or:

$$g=\frac{w_{0}}{1-w_{0}}{\left(u_{0-}u\right)}^{2}$$ (4)

For each gray level *L*, let *t* vary to update the *w*_*i*_ and *u*_*i*_, such that the inter-class variance *g* achieved its maximum value, and the corresponding *t* of the maximized *g* was the optimal threshold for the image. The Otsu method for image segmentation was carried out in IDL 8.5.

#### 2.4.2 Selection of a vegetation index

There are many vegetation indices that can be used to estimate the FVC from RGB images; the conditions under which each of them is most effective are different. Therefore, we performed a test to determine the optimal vegetation index for our study area. After consulting relevant literature, we selected three vegetation indices (Table 1) with superior performance in distinguishing between vegetation and non-vegetation [30–32], and evaluated their performance on the RGB images of our study area. The indices were: Excess Green minus Excess Red Index (ExGR), Excess Green Index (ExG), and Woebbecke index (WI).

**Table 1:** Comparison of vegetation indices.

Vegetation index	Definition	FVC	Reference
ExGR (Excess Green minus Excess Red Index)	(3g-2.4r-b)	24.52%	[33]
ExG (Excess Green Index)	2g-r-b	17.48%	[34]
WI (Woebbecke index)	(g-b)/(r-g)	15.75%	[34]

r, g and b are the pixel values from the images based on each RGB channel (red channel/green channel/blue channel).

After calculating the three vegetation indices, we used the Otsu method to determine the vegetation and non-vegetation thresholds and calculated the corresponding FVC values. Two methods were used to verify the accuracy of the vegetation indices: (1) the Support Vector Machine (SVM) method in ENVI 5.3 to classify and calculate the FVC of the test image; (2) using the ArcGIS 10.2 software to randomly generate 400 samples from the original image and visually interpreting whether each pixel was a vegetation pixel.

**Fig 3:**
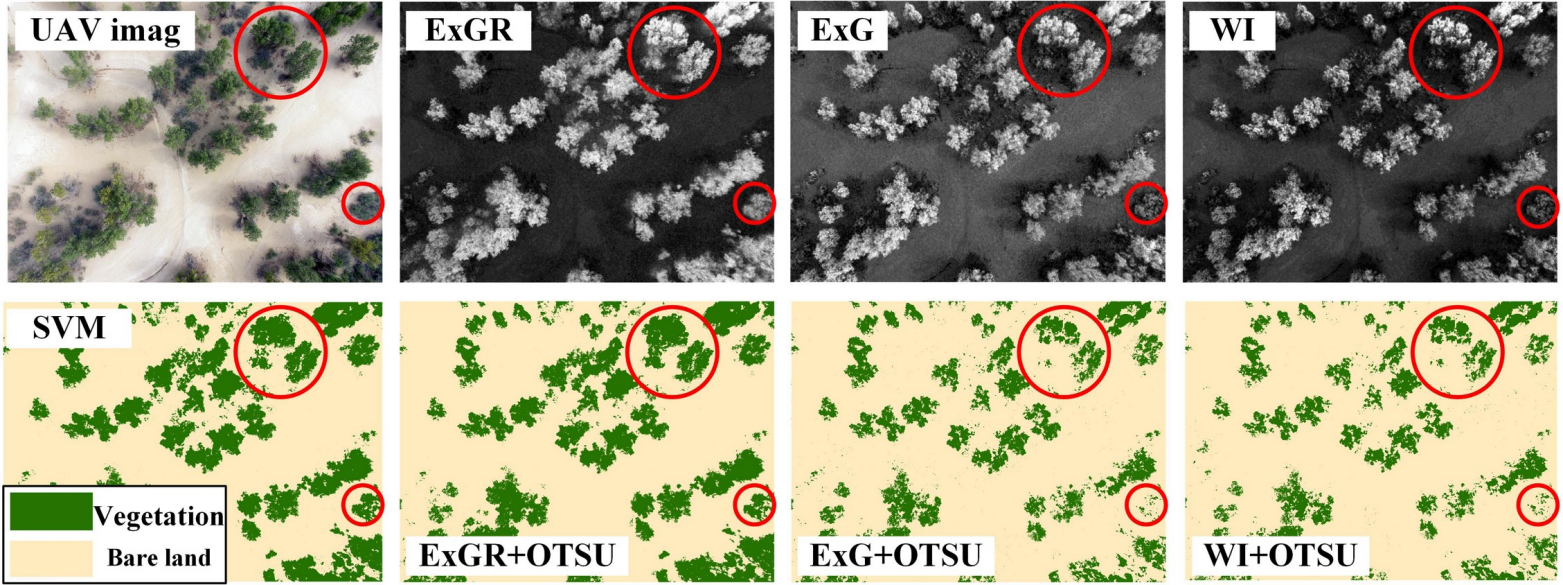
The vegetation extraction performance of the three vegetation indices. The vegetation identified by each index is marked with a red ring. Abbreviations: ExG, the Excess Green Index; ExGR, Excess Green minus Excess Red Index; OTSU, the Otsu threshold segmentation method; SVM, Support Vector Machine; UAV, unmanned aerial vehicle; WI, the Woebbecke index.

From the three vegetation indices tested, the ExGR had the best separation effect and extracted the most complete vegetation information (Fig 3). The FVC estimated by the three vegetation indices was 24.52% (ExGR), 17.48% (ExG), and 15.75% (WI). The FVC of the test image estimated by the two verification methods was 25.46% for verification method (1), while verification method (2) showed that among the 400 randomly sampled points, there were 101 vegetation pixels and 299 non-vegetation pixels (FVC of 25.25%). Therefore, on the basis of these results, we selected the ExGR index for estimating the FVC.

### 2.5 Analysis of the frequency distribution of the FVC

The Gaussian function is also known as the normal distribution function, and many ecological data can be described by it [35]. We used the Gaussian function to fit the frequency curve of the FVC to the sample set, because it produced a clearer visual representation of the influence of quadrat size on the sampling result. The Gaussian function can be expressed as:

$$f(x)=ae^{-\frac{{\left(x-\mu \right)}^{2}}{2\sigma^{2}}}$$ (5)

where *a* is the peak of the fitted curve, *μ* is the mean, *σ* is the standard deviation. *R*^*2*^ represents the correlation coefficient of the Gaussian function when fitted to the frequency curve.

First, we used OriginPro 2016 to count the frequency of FVCs and plot the frequency distribution histogram. Next, using the Gaussian function (performed using the analysis tool of OriginPro 2016) to fit the frequency curve of the sample FVCs. Finally, we analyzed the changing characteristics of frequency curves created with different quadrat sizes.

### 2.6 Determining the optimal quadrat size

The mean and variance of the samples are important indicators that can be used to judge the efficacy of the sampling unit. Thus, we used Microsoft Excel 2016 to calculate the mean and variance of the FVC of the samples under different quadrat sizes, and using OriginPro 2016, we drew scatter diagrams of quadrat size versus FVC variance and then fit quadrat size-FVC variance curves. When the variance reaches a minimum, it indicates that the corresponding quadrat size gives the best sampling results, and that quadrat size (inflection point) can be taken as the optimal one.

## 3. Results

### 3.1 Frequency distribution features of the FVC

**Fig 4:**
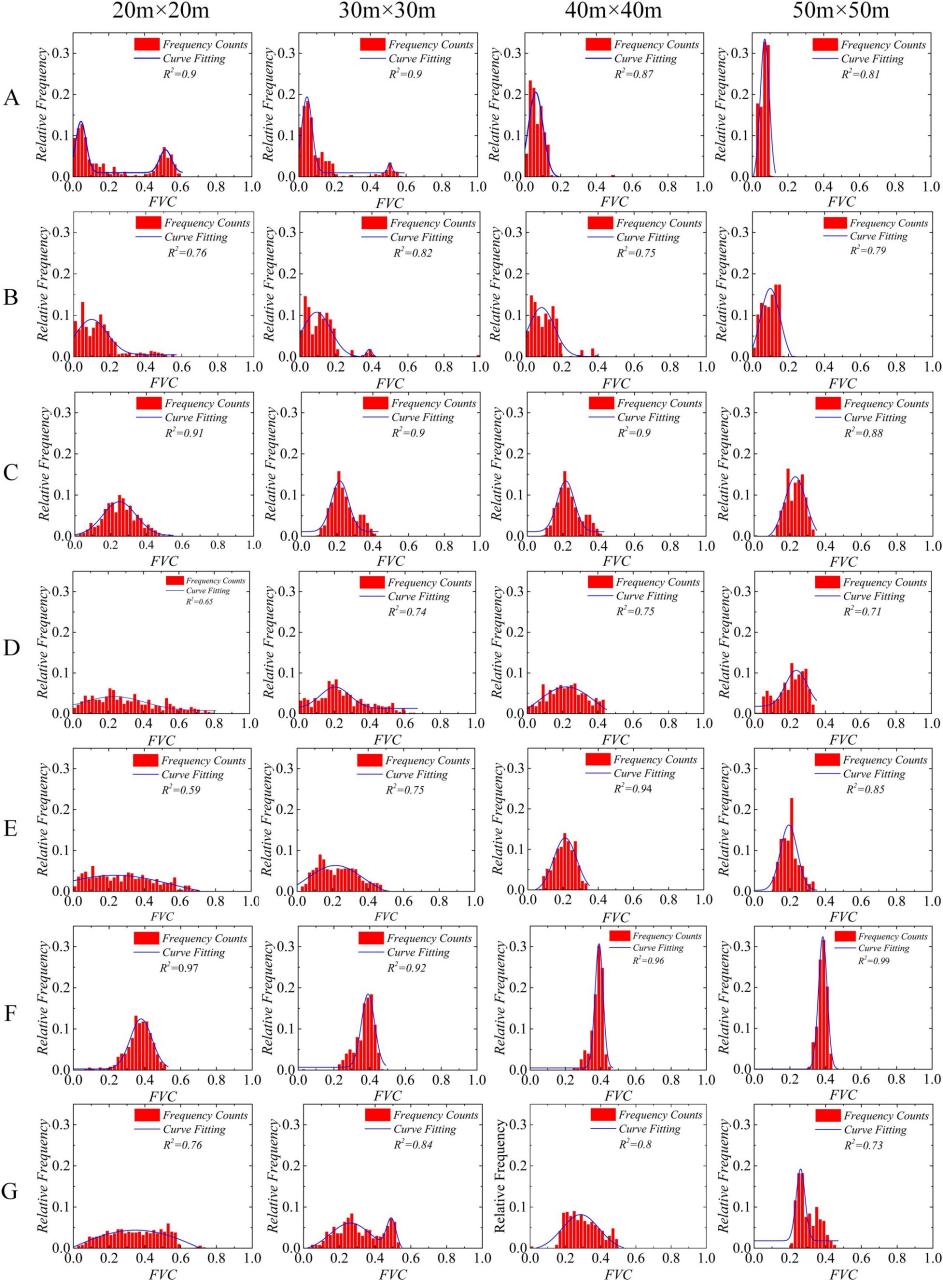
The fractional vegetation coverage (FVC) index frequency distribution in the study plots depended on quadrat size. Quadrat sizes are 20 m × 20 m, 30 m × 30 m, 40 m × 40 m, and 50 m × 50 m; the sample size under each quadrat size is 500 (n = 500). R^2^ represents the correlation coefficient of the fitted curve. A, B, C, D, E, F, and G are the study plots.

The FVC calculated from the original UAV image size (100 m × 100 m) was defined as the true FVC. In plot A, when the quadrat size was 20 m × 20 m, the R^2^ of the fitted curve was more than 0.8, the fitting curve had two peaks, the mean values (*μ*) were 0.044 and 0.52, the variance (*σ*^*2*^) values were 0.029 and 0.033, and the relative frequency values were 0.14 and 0.07 (Fig 4). The FVC sampling results in plot A were mainly concentrated around 0.044 and 0.52, with a large range of variation. The sampling results were not robust and had a large deviation from the true value of FVC (FVC_*true*_ = 0.05) in plot A. This implies that when a quadrat size of 20 m × 20 m is adopted for field vegetation surveys in areas that are similar to this plot, the quadrats were too small to cover both the sparse and dense vegetation areas. Since a given quadrat may happen to exclusively contain sparse or dense vegetation, this can lead to the occurrence of extrema; thus, this quadrat size is likely to skew the sampling results, making them unlikely to be representative. The frequency distribution of the FVC gradually concentrated around the true value as quadrat size increased (Fig 4). When the quadrat size was 50 m × 50 m, the μ of the samples was 6.67%, which was relatively close to the true value of the FVC (0.05), and the variance *σ*^*2*^ was 0.022. According to the frequency count, 79.6% of the FVC sampling results were distributed within 3–9%.

The frequency distribution features of the FVC in plot E were significantly different from those in plot A. Under a quadrat size of 20 m × 20 m, the FVC sampling results were evenly distributed between 0 and 0.68, with a wide range of variation and a relatively low frequency with a maximum of only 7%. This indicates that adopting a quadrat size of 20 m × 20 m, when carrying out field vegetation surveys in areas with similar characteristics to this plot would lead to strong fluctuations in the sampling results; thus, this quadrat size was not representative. As quadrat size increased, the *σ*^*2*^ of the FVC gradually decreased, and the frequency distribution gradually concentrated within a smaller range. When quadrat size was 50 m × 50 m, the *μ* was 0.2, the *σ*^*2*^ was 0.02, and the maximum of the relative frequency was 23%. According to the frequency count, 77.8% of the FVC sampling results were distributed within 16%-32%, which is significantly better than the result from a quadrat size of 20 m × 20 m.

In plot F, with the increase in quadrat size, the maximum frequency of the FVC increased gradually as follows: 13.2% (20 m × 20 m), 18.4% (30 m × 30 m), 30.2% (40 m × 40 m), 31.6% (50 m × 50 m), and 31.6% (60 m × 60 m). This shows that the sampling results tended to be relatively stable and only gradually increase with quadrat size; however, the *μ* and *σ*^*2*^ of FVC varied slightly (Table 2).

**Table 2:** The mean (*μ*) and variance (*σ*^*2*^) of the sample fractional vegetation coverage (FVC) index.

Quadrat (m^2^)	Plot A		Plot B		Plot C		Plot D		Plot E		Plot F		Plot G	
	*u*	σ^2^	*u*	σ^2^	*u*	σ^2^	*u*	σ^2^	*u*	σ^2^	*u*	σ^2^	*u*	σ^2^
10×10	0.355	0.055	0.246	0.037	0.340	0.026	0.434	0.052	0.414	0.035	0.362	0.012	0.388	0.028
20×20	0.223	0.044	0.142	0.013	0.253	0.009	0.305	0.035	0.282	0.030	0.367	0.005	0.343	0.023
30×30	0.115	0.020	0.117	0.010	0.237	0.005	0.246	0.020	0.227	0.012	0.370	0.003	0.315	0.016
40×40	0.068	0.002	0.104	0.005	0.228	0.003	0.223	0.011	0.208	0.003	0.379	0.001	0.299	0.008
50×50	0.067	0.000	0.096	0.002	0.228	0.002	0.206	0.006	0.201	0.002	0.382	0.001	0.300	0.003
60×60	0.067	0.000	0.094	0.004	0.231	0.002	0.215	0.004	0.202	0.003	0.374	0.001	0.303	0.001

FVC, fractional vegetation coverage; μ represents the mean; σ^2^ represents the variance. Sample size n = 500.

The ideal result of a field vegetation survey is a sample value that is concentrated within a small range, and a high frequency, in which case the sampling results are representative and can reflect the overall vegetation characteristics well. We conducted the sampling simulation in plots with different distribution characteristics. Our results showed that the sampling results tended to be gradually more stable and approach their true values as the quadrat size increases. However, while theoretically a larger sample area improves the sampling design, this is often not practically achievable. Therefore, to determine the optimal quadrat size, we needed to further observe how the variation in *μ* and *σ*^*2*^ changed with increasing quadrat area.

### 3.2 Variation rate of σ^2^

**Fig 5:**
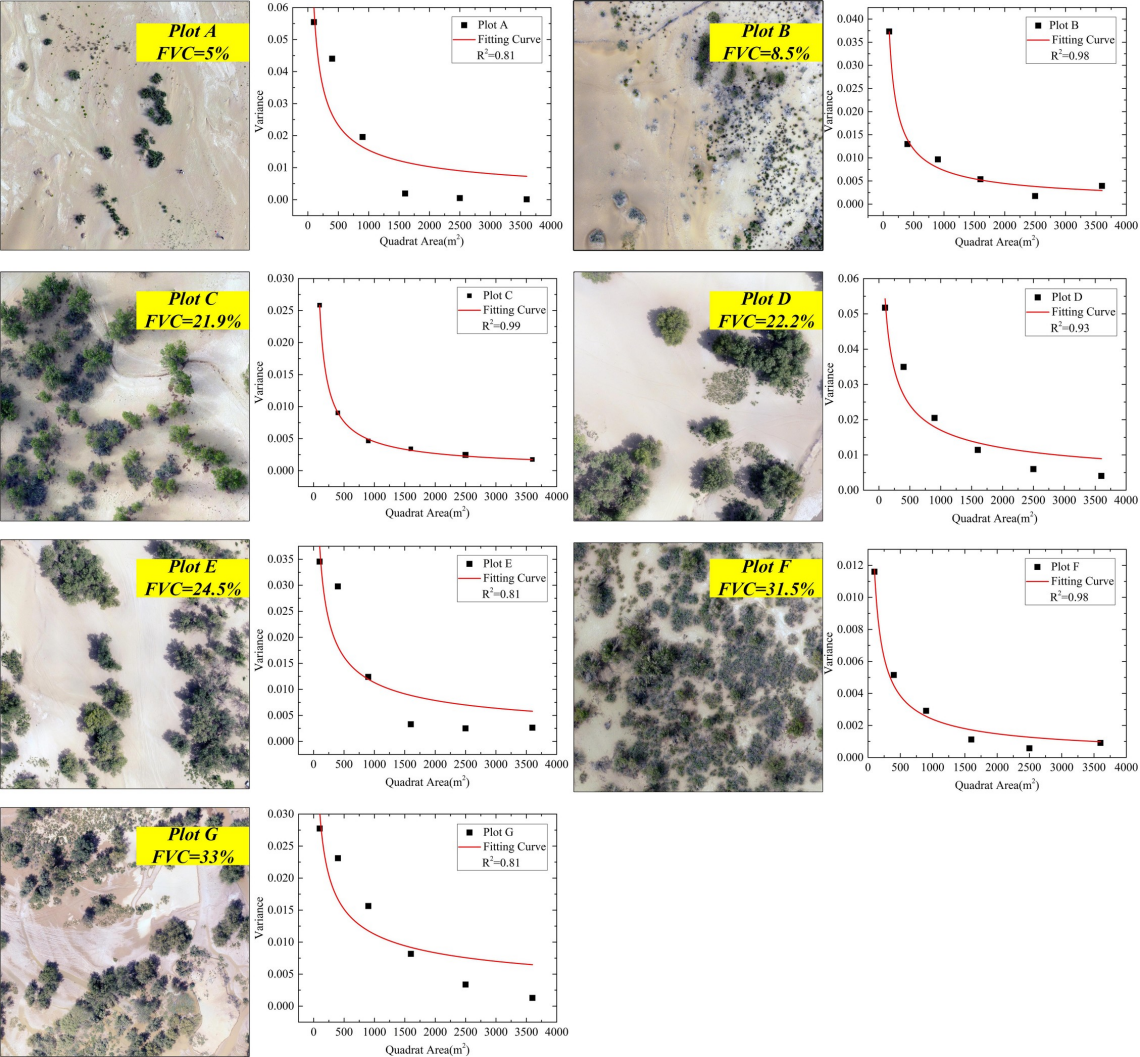
The changing trend of the variance of the sample fractional vegetation coverage (FVC) index with different quadrat sizes. The figure shows the changing features of the variance *σ*^*2*^ (n = 500) in seven study plots (A–G, each plot 100 m × 100 m) with different quadrat sizes. The horizontal axis represents quadrat size (m^2^), and the vertical axis represents the *σ*^*2*^ obtained by random repeating sampling 500 times with a fixed quadrat size.

The maximum *σ*^*2*^ seen in the seven test plots was 0.055, when the quadrat size was 10 m × 10 m (Fig 5). As the quadrat size increased, *σ*^*2*^ decreased severely in the range of 10 m × 10 m to 30 m × 30 m. Although the quadrat size was between 30 m × 30 m and 50 m × 50 m, the variation rate in *σ*^*2*^ tailed off. At the size of 50 m × 50 m, it approached its minimum value, indicating that any further enlargement of quadrats provided no significant improvement in the sampling results. Among the seven test plots, vegetation in plots A, B, D, and E was sparse and unevenly distributed. It is therefore the spatial heterogeneity of the vegetation that caused the strong variation in the observed FVC. In plots C, F, and G, the variation rate of *σ*^*2*^ varied only slightly, due to the relatively even distribution of vegetation. In terms of vegetation coverage, the lower the coverage, the more severe the variation.

All the random sampling simulations of the different test plots showed the same pattern: *σ*^*2*^ and its variation rate both decreased with increases in quadrat size. When quadrat size was 50 m × 50 m, the variation rate of *σ*^*2*^ changed very slightly with the further enlargement of the quadrat, showing that 50 m × 50 m was the appropriate quadrat size for this field survey. Thus, we conclude that it is reasonable and feasible to take the inflection point of the variation rate of *σ*^*2*^ as the optimal quadrat size for field vegetation surveys.

## 4. Discussion

### 4.1 Changes in μ and σ^2^

In our sampling simulations, the frequency distribution of the FVC and its fitted curve varied with increases in quadrat size. When quadrat size increased, the *σ*^*2*^ decreased correspondingly, along with the occurrence of extreme values. When quadrat area is small, extrema were more likely to occur in the samples, and the *σ*^*2*^ of the samples was large. In some plots, both *μ* and *σ*^*2*^ decreased as quadrat size increased, while in other plots, only *σ*^*2*^ showed this effect, while *μ* did not decrease significantly (Table 2). Regarding the disappearance of the extrema and the variation in *μ* and *σ*^*2*^, Bellehumeur (1997) thought that the value of a small quadrat was absorbed as a component of the large quadrat, which meant that the extrema would manifest in a large quadrat. Therefore, the value of *μ* remains unchanged while *σ*^*2*^ decreases.

In desert areas, sparse, unevenly distributed vegetation results in samples displaying high variance [36]. Previous studies have illustrated that increasing the quadrat size can decrease this variance [37]. In these studies, variance was used as a key indicator when estimating the optimum quadrat size [38, 39]. Mosley (1989) considered that a quadrat size that easily leads to the occurrence of an extremum is not suitable as a sampling unit. The results of our simulations are consistent with the finding of Bellehumeur (1997), that the enlargement of quadrats can effectively suppress the *σ*^*2*^ of samples and reduce the frequency of extrema. A large quadrat is more representative in vegetation surveys, but when the quadrat area was expanded beyond a certain size, the *σ*^*2*^ of samples did not decrease much further. This indicates that at that point, the quadrat area is sufficient to effectively filter the differences in the small-range distribution of vegetation, and that this quadrat size is appropriate for the sampling survey.

### 4.2 Method validity

The application of our methodology to the Daliyaboyi Oasis showed that when the quadrat size was 50 m × 50 m, the frequency distribution of the FVC was concentrated within a small range, and the *σ*^*2*^ of samples approached its minimum value. Our conclusion that 50 m × 50 m is the appropriate quadrat size for vegetation surveys at this site is validated by previous studies conducted in very similar conditions. Specifically, the ecosystem of the Daliyaboyi Oasis is similar to the lower reaches of the Tarim River, with the plant communities in both locations dominated by *Populus euphratica* and *Tamarix chinensis*. Li, Tang [40] investigated 38 quadrats of two sections in the lower reaches of the Tarim River and found that the accuracy of plant species surveys could reach 90% with a quadrat size of 64 m × 64 m. Niu, Xu [41] also investigated the lower reaches of the Tarim River; their results showed that the quadrat sizes that met 90% of the survey accuracy at the three sections investigated were 2500 m^2^, 3600 m^2^, and 4500 m^2^, respectively. This similarity in the results achieved using different methods lends support to the methodology proposed by our study.

### 4.3 Method strengths and limitations

In ecological studies, both remote sensing data and ground truth data are required. The ground truth data can verify the accuracy of remote sensing data, but more often, ground data and remote sensing data together constitute data sets to solve ecological problems. However, due to the shelter effect of the plant canopy, it is difficult to obtain some vegetation parameters by remote sensing, such as the number of trees, diameter at breast height (DBH), first branch height. In addition, some parameters can only be measured manually by using specific instruments or tools, such as collecting plant leaves to detect their nutrient content, the number of seeds, and the measurement of soil moisture. Therefore, selecting an appropriate quadrat size for performing ground surveys is imperative. Meanwhile, UAV technology plays an increasingly important role in vegetation studies, and has been widely applied in vegetation mapping, plant conservation, retrieval of vegetation structural parameters and other studies [42–45]. It is necessary to effectively combine remote sensing data and in-situ data to better perform vegetation studies [46].

In fieldwork, it is often difficult to establish a large sample size based on measured data that could then be used to estimate the appropriate quadrat size. Besides, desert areas are especially notorious for their harsh climate and poor transportation, making them difficult to research. The application of UAV technology can help researchers to overcome severe weather conditions and investigate complex terrains, while retaining full control of spatial resolution. We believe that the use of UAV to acquire high-precision images of study areas for random sampling simulations is a significant strength of our methodology, which can thus be applied to other desert areas to estimate optimum quadrat size with limited labor, thus improving fieldwork efficiency, and achieving high survey accuracy. In addition, the simulation covers multiple sampling possibilities, and has good stability, which is conducive to acquiring accurate sampling results. Thus, this study exploited the advantages of UAV to determine the optimal quadrat size for ground surveys, which is an attempt at a partially UAV-assisted ground survey.

In this study, our objective was to propose a methodology for choosing a suitable sampling scale for obtaining accurate sampling estimation in areas with sparse vegetation cover, and test it in a desert study site. We specifically aimed to overcome the difficulties in vegetation survey that are caused by the discontinuous distribution of vegetation, which if unaddressed can lead to large discrepancies in survey results. These difficulties include the dependence of vegetation surveys on vegetation spatial distribution patterns, and the potential under- or overestimation of sampling requirements. If appropriately selected, the right quadrat size will match the characteristics of such sites and overcome the differences caused by the clumped vegetation distribution, benefitting the overall estimation. However, it must be noted that our study aimed to demonstrate the suitability of our proposed methodology, using the Daliyaboyi Oasis as a test site; therefore, 50 m × 50 m size should not be taken as the default best quadrat size for other locations. Instead, to account for the spatial heterogeneity and the unique characteristics of each site, we propose that the appropriate quadrat size for other desert areas with sparse vegetation should be determined by this method. In addition, we adopted FVC as a suitable metric for determining the optimum survey quadrat; this method for determining optimal quadrat size is only suitable for surveys of sparse-vegetation areas such as deserts. This conclusion is based on the observation that the variation in FVC in a high FVC area is very slight, and therefore cannot be used to determine the appropriate quadrat size.

## 5. Conclusions

To address the issue of determining the optimal quadrat size for surveys in desert areas with sparse vegetation, we proposed a method based on low-altitude UAV images and simulated random sampling. The simulated random sampling was used to establish the sample sets and calculate their FVC. This was followed by an analysis of the frequency distribution and the variation in FVC, which facilitated determination of the appropriate quadrat size for our study area. Compared with the traditional methods, our methodology is both simpler and more robust. It can effectively reduce the field workload, provide quantitative references for the design of field vegetation surveys, and effectively improve the efficiency and accuracy of field vegetation investigations.

## Supporting information

S1 File The IDL code of calculates FVC from RGB images in batch. (ZIP)

S1 Data The sample FVC of seven test plots. (XLSX)
